# Unexpected positive intraoperative cultures (UPIC) at index osseointegration do not lead to increased postoperative infectious events

**DOI:** 10.5194/jbji-7-155-2022

**Published:** 2022-07-18

**Authors:** Jason S. Hoellwarth, Taylor J. Reif, Michael W. Henry, Andy O. Miller, Austin C. Kaidi, S. Robert Rozbruch

**Affiliations:** 1 Limb Lengthening and Complex Reconstruction Service, Hospital for Special Surgery, Weill Cornell Medical College, 535 East 70th Street, New York, NY 10021, USA; 2 Infectious Disease Service, Hospital for Special Surgery, Weill Cornell Medical College, 535 East 70th Street, New York, NY 10021, USA

## Abstract

**Introduction**: The most common complication following transcutaneous osseointegration for amputees is infection. Although an obvious source of contamination is the permanent stoma, operative site contamination at the time of implantation may be an additional source. This study investigates the impact of unexpected positive intraoperative cultures (UPIC) on postoperative infection. **Methods**: Charts were reviewed for 8 patients with UPIC and 22 patients with negative intraoperative cultures (NIC) who had at least 1 year of post-osseointegration follow-up. All patients had 24 h of routine postoperative antibiotic prophylaxis, with UPIC receiving additional antibiotics guided by culture results. The main outcome measure was postoperative infection intervention, which was graded as (0) none, (1) antibiotics unrelated to the initial surgery, (2) operative debridement with implant retention, or (3) implant removal. **Results**: The UPIC vs. NIC rate of infection management was as follows: Grade 0, 6/8 = 75 % vs. 14/22 = 64 %, *p* = 0.682; Grade 1, 2/8 = 25 % vs. 8/22 = 36.4 % (Fisher's *p* = 0.682); Grade 2, 1/8 = 12.5 % vs. 0/22 = 0 % (Fisher's *p* = 0.267); Grade 3, 0/8 = 0 % vs. 1/22 = 4.5 % (Fisher's *p* = 1.000). No differences were statistically significant. **Conclusions**: UPIC at index osseointegration, managed with directed postoperative antibiotics, does not appear to increase the risk of additional infection management. The therapeutic benefit of providing additional directed antibiotics versus no additional antibiotics following UPIC is unknown and did not appear to increase the risk of other adverse outcomes in our cohort.

## 1 Introduction

When performing elective reconstructive orthopedic surgery involving implantation of large permanent metal implants, it is generally considered optimal to have a patient and local wound environment without infection in order to optimize wound healing and infection risks. There is substantial literature investigating the significance of unexpected positive intraoperative cultures (UPIC) identified during surgery for primary total hip, knee, and shoulder replacement (Zmistowski et al., 2021; Wong et al., 2018; Jonsson et al., 2014; Ferro et al., 2020), revision total joint implantation (Padegimas et al., 2017; Hipfl et al., 2021; Pérez-Prieto et al., 2021), and open fractures (Agrawal et al., 2013).

Transcutaneous osseointegration, a relatively recent reconstructive rehabilitative option for amputees (Hoellwarth et al., 2020), is receiving increasing attention and is different from other prosthetic reconstruction scenarios for several reasons. The primary distinction is that a transcutaneous permanently open skin stoma remains, through which the skeletally anchored metal implant is attached to the external prosthetic limb. This skin interruption is a site of potential frequent and direct bacterial ingress which can colonize or infect the soft tissues, bone, and implant. Unsurprisingly, the most common adverse event following osseointegration is infection (Reif et al., 2021), which may require oral antibiotics, operative debridement with implant retention, or implant removal with additional debridement (Al Muderis et al., 2017). Prior investigations of post-osseointegration infection have reported postoperative occurrence rates and management (Al Muderis et al., 2017; Reif et al., 2021; Hoffmeister et al., 2017; Atallah et al., 2020). However, it is currently unknown to what extent UPIC impacts the risk of subsequent infection.

To investigate that question, this study compared the infection-related adverse events of two cohorts of osseointegrated amputees: those with UPIC versus those with negative intraoperative cultures (NIC). The primary outcome was whether patients eventually had any intervention for a postoperative infectious concern (antibiotics or surgery).

## 2 Methods

Following institutional ethics approval, all 55 patients in our prospectively maintained osseointegration registry were retrospectively evaluated. Included patients met the following criteria: index osseointegration performed at least one year prior to this study (October 2017 to October 2020) ( n=37 ), with intraoperative cultures taken. Seven patients did not have intraoperative cultures taken: two because they had primary amputation with simultaneous osseointegration (not already an amputee), so they were not considered a potential risk for latent infection, and five others inadvertently did not have cultures taken. These seven were excluded from the study, yielding 30/37 (81 %) of potentially eligible patients who were evaluated. All patients had at least 1 year of follow-up.

Charts were reviewed for the following perioperative information: demographic data, the results and sensitivity of intraoperative cultures, the immediate postoperative antibiotic regimen for patients with UPIC, any relevant prior surgery, and any postoperative infection-related management. The operative routine was to take five culture swabs prior to implant insertion, and if at least one of these swabs resulted positive, to provide antibiotics as guided by the infectious disease consultants. The main outcome of postoperative infection management was graded as zero to three, defined in Table 1.

**Table 1:** Grading system for infection-related management of osseointegrated patients.

Grade	Features	Associatedmanagement
0	No infectious features	None
1	Stoma erythema and/ortenderness and/or minor leakage	Oral antibiotics
2	Stoma leakage and/or painful weightbearing and/or sinus tract and/or radiographic peri-implant osteolysis	Operative soft tissue and/or bone debridement, implant retention
3	Same as Grade 2 and/or gross implant motion	Operative debridement, implant removal

Frequency comparison was performed using Fisher's exact test. Means were compared using Student's t test. Significance was defined as p≤0.05 .

### 2.1 General osseointegration consideration and technique

In general, amputees are offered osseointegration if they express dissatisfaction with their socket prosthesis in regards to fit, pain, mobility, or overall quality of life. Patients with an intact limb are offered amputation with osseointegration if they have complex deformity or pain for which an amputation is expected to provide functional improvement. We perform osseointegration with custom-ordered press-fit titanium implants featuring porous coating at the bone interface and a smooth surface at the skin interface (Osseointegrated Prosthetic Limb, Permedica Medical Manufacturing, Lecco, Italy; and Signature Orthopaedics, New South Wales, Australia). General contraindications to osseointegration include active disease which puts healing at risk, such as active infection. Patients who appear to have active infection are provided a disinfection surgery which debrides nonviable soft tissue and bone and places a local antibiotic depot, which is followed by a recovery period of approximately 6 or more weeks. If the patients have physical examination and laboratory markers consistent with infection eradication, osseointegration may be provided. We do not place osseointegration implants if there is any concern or perceived risk for a contaminated wound bed. We routinely take five culture swabs from the intramedullary canal upon initial preparation, prior to reaming, which are incubated for aerobic (5 days) and anaerobic (14 days) bacteria. The first three patients had two-stage osseointegration approximately 2 months apart, but the remainder had single-stage surgery. All incisions are primarily closed leaving the stoma surrounding the transcutaneous dual cone prosthesis adapter. Patients are admitted postoperatively for approximately 3–5 days for pain control, early rehabilitation, and stoma self-care education. Routine perioperative antibiotics (weight-based cefazolin unless contraindicated) are continued for 24 h postoperatively. For patients whose intraoperative cultures result positive, an antibiotics course is constructed in consultation with infectious disease physicians. Patients are routinely evaluated by the surgeon with radiographic and physical examination at 3 weeks, 3 months, 6 months, and annually after osseointegration. At each office visit, we remind patients that if they experience symptoms concerning for infection, they should directly inform our office, rather than a local doctor or emergency department, in order to minimize antibiotic over-prescription or under-diagnosis.

## 3 Results

The patient demographic summary is presented in Table 2. The following comparisons were statistically different. UPIC had a greater proportion of right-sided surgery and traumatic etiology for amputation than NIC. Cohorts were not statistically different regarding age at osseointegration, age at initial amputation, gender distribution, height, weight, tobacco use, stages of osseointegration, implant used, implant diameter or length, erythrocyte sedimentation rate (ESR), C-reactive protein (CRP), or prior staged disinfection surgery. No patients had remnant orthopedic hardware at the time of osseointegration, other than the one who had an antibiotic spacer placed in preparation for osseointegration.

**Table 2:** Patient demographics, organized by operative culture status. Boldface type indicates statistical significance.

Variable	UPIC ( n=8 )	NIC ( n=22 )	$p{=}^{*}$
Age at osseointegration (years)	54.6±12.7	46.9±13.0	0.173
Age at amputation	47.2±16.5	37.3±13.4	0.159
Female	1 (12.5 %)	8 (36.3 %)	0.374
Height (cm)	177±8.6	173±9.1	0.302
Weight (kg)	82.4±18.9	90.4±22.9	0.346
Right side	1 (12.5 %)	13 (59.1 %)	**0.040**
Trauma etiology	4 (50 %)	20 (90.1 %)	**0.029**
Tobacco use	1 (12.5 %)	1 (4.5 %)	0.469
Single stage	7 (87.5 %)	20 (90.1 %)	1.000
OPL brand	8 (100 %)	21 (95.5 %)	1.000
Implant diameter (mm)	17.3±5.1	18.8±4.1	0.725
Implant length (mm)	129.4±21.8	131.4±35.5	0.856
ESR ≥15	1 (12.5 %)	5 (22.7 %)	1.000
CRP ≥1	2 (25 %)	5 (22.7 %)	1.000
Staged disinfection surgery	0 (0 %)	4 (18.2 %)	0.550

UPIC – cohort with unexpected positive intraoperative cultures. NIC – cohort with negative intraoperative cultures. ESR – erythrocyte sedimentation rate; our institution considers 15 or greater abnormal. CRP – C-reactive protein; our institution considers 1 or greater abnormal. ${}^{*}$ The techniques for frequency and means comparison are described in the Methods section.

Table 3 profiles the UPIC patients along with their subsequent antibiotic regimens. All patients had antibiotic therapy organized by the infectious disease consultants. The duration of all treatment regimens was 6–8 weeks. No patients experienced major adverse effects such as *Clostridium difficile* colitis.

**Table 3:** Summary of patients with UPIC.

Patient no.	Age sex bone	Etiology ofamputation	Cultured bacteria*	Treatment regimen
1	47 M humerus	Electrocution	*Propionibacterium acnes* (1/5)	Clindamycin (300 mg, oral twice daily, 6 weeks)
2	26 M femur	Trauma	*Staphylococcus capitis subspecies ureolyticus* (1/6)	Sulfamethoxazole–trimethoprim (800–160 mg, oral twice daily, 12 weeks)
3	60 M femur	Infection	*Staphylococcus epidermidis* (2/5)	Sulfamethoxazole–trimethoprim(800–160 mg, oral twice daily, 6 weeks)
4	56 F tibia	Trauma	*Staphylococcus epidermidis* (3/3)	Vancomycin (red man syndrome) switched to daptomycin (rhabdomyolysis) switched to doxycycline (100 mg, oral twice daily, 6 weeks)
5	60 M tibia	Trauma	*Enterococcus casseliflavus* (3/3); *Stenotrophomonas (Xanthomonas) maltophilia* (3/3)	Daptomycin (500 mg, intravenous daily, 12 weeks) along with levofloxacin (750 mg, oral daily, first 6 weeks) followed by amoxicillin (875 mg, oral daily, second 6 weeks) and sulfamethoxazole–trimethoprim (800–160 mg, oral daily, second 6 weeks)
6	66 M femur	Trauma	*Finegoldia magna * (5/5)	Ertapenem (1000 mg, intravenous daily, 8 weeks)
7	60 M tibia	Deformity	*Staphylococcus epidermidis* (5/5)	Daptomycin (500 mg, intravenous daily, 6 weeks
8	58 M femur	Infection	*Proteus mirabilis* (5/7); *Klebsiella pneumoniae* (4/7); *Morganella morganii* (2/7)	Ciprofloxacin (750 mg, oral twice daily, 6 weeks)

${}^{*}$ Following each bacteria, the parentheses identify the number of cultures which were positive for this bacteria/the number of cultures that were taken.

Table 4 presents the postoperative infection-related events. All oral antibiotics were prescribed in response to the clinical appearance of the stoma or skin. The rate of oral antibiotic prescription for UPIC patients was 2/8 = 25 %; for NIC patients it was 8/22 = 36.4 % (Fisher's p=0.682 ). The UPIC vs. NIC rate of debridement was 1/8 = 12.5 % vs. 0/22 = 0 % (Fisher's p=0.267 ), and for implant removal it was 0/8 = 0 % vs. 1/22 = 4.5 % (Fisher's p=1.000 ), neither a significant difference.

**Table 4:** Infection-related events after osseointegration.

Cohort	UPIC n=8				NIC n=22			
Grade	0	1	2	3	0	1	2	3
Humerus n=2	1	0	0	0	1	0	0	0
Femur n=17	4	0	0	0	6	7	0	0
Tibia n=11	1	1 (2)	1	0	7	0	0	1
Total n=30	6	1 (2)	1	0	14	7 (8)	0	1

The number in each cell identifies the number of patients who had at most that grade of infectious management. The parenthetical number ( x ) indicates the total number of patients who were provided that level of intervention (such as oral antibiotics) but eventually escalated to a greater degree of intervention (such as debridement or implant removal). Specifically, 3 transtibial patients had UPIC, 2 patients were provided additional oral antibiotics of which 1 progressed to having irrigation and debridement; 8 transfemoral patients had UPIC, 2 patients were provided additional oral antibiotics of which 1 progressed to having irrigation and debridement; 22 transfemoral patients had NIC, 7 were provided additional oral antibiotics of which 1 progressed to having the implant removed.

**Figure 1:**
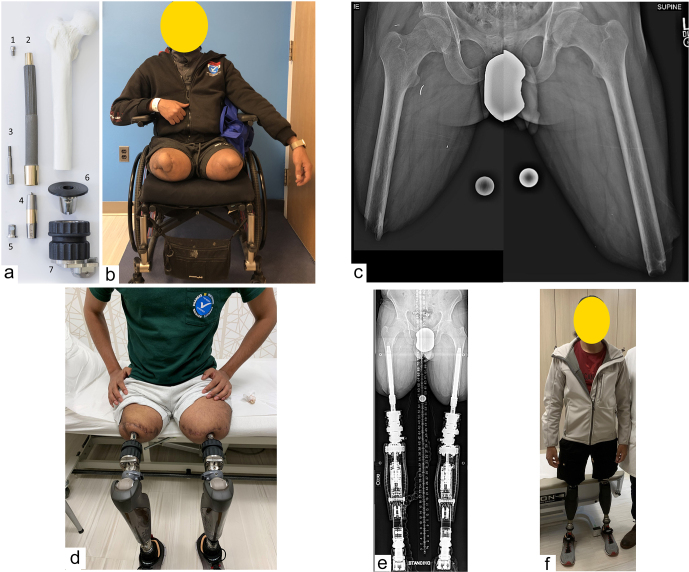
Osseointegration implant and clinical patient photograph. The Osseointegrated Prosthetic Limb (OPL) which was the implant used for nearly every patient in this study. It is a forged titanium alloy, stem-shaped implant whose surfaces have a plasma-sprayed coating, up to 0.5 mm thick, to promote bone ingrowth and rapid integration. The external portions of the collars are treated with titanium niobium oxynitride ceramic to promote smooth soft-tissue gliding, limiting the probability of symptomatic soft-tissue adhesion and tethering. Proximal fluted fins provide initial rotational stability, akin to a Wagner-style hip arthroplasty stem. **(a)** Exploded view with the components arranged at approximately the proximal–distal levels in which they would be once assembled and implanted in a patient who had undergone a femoral amputation: (1) proximal cap screw; (2) OPL body; (3) safety screw; (4) dual cone abutment adapter; (5) permanent locking propeller screw; (6) proximal connector; and (7) prosthetic connector. **(b)** Photograph of a 28-year-old male with bilateral transfemoral amputations, requiring a wheelchair for locomotion. **(c)** Preoperative left and right femur radiographs, assembled to portray patient's preoperative osteology. **(d)** Three months following osseointegration, the patient was fit with bilateral prosthetic legs. Note the transcutaneous nature of the skeletally linked prostheses. **(e)** Long standing radiographs of the patient with the osseointegrated implants connected to the prosthetic legs. Note that unlike many transfemoral amputees using a socket prosthesis whose hip joints are abducted against the socket liner, this patient's femurs are anatomically oriented with the hip, knee, and ankle in excellent mechanical alignment. **(f)** Photograph of patient standing without a walking aid 1 year after osseointegration.

**Figure 2:**
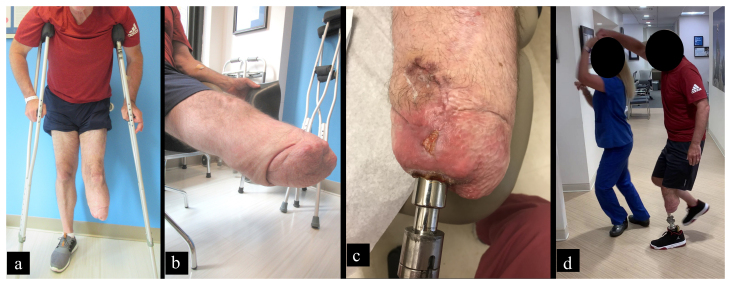
UPIC patient who had additional surgery to manage infection. **(a)** Clinical photograph identifying the patient was unable to wear his prosthesis before surgery and was relegated to crutch ambulation because **(b)** the socket prosthesis caused painful skin ulcers. **(c)** He developed a sinus tract which was debrided 9 months after the index surgery. **(d)** Within 5 months, the patient was able to return to a higher level of activity than before osseointegration, seen here demonstrating the ability to plant on his osseointegrated leg in order to turn a dance partner.

Two patients had additional surgery to manage infectious issues. One transtibial UPIC patient (Patient 5) had a draining sinus tract near the skin closure, without pain, without radiographic osteolysis. Nine months after index osseointegration, he had irrigation and debridement of the sinus tract, soft tissue, and a minimal amount of unhealthy appearing bone, retaining the implant. His cultures at index osseointegration grew *Enterococcus casseliflavus; Stenotrophomonas (Xanthomonas) maltophilia *and he had the antibiotic regimen as reported in Table 3. His debridement cultures grew *Pseudomonas aeruginosa* (3/5), *Staphylococcus epidermidis* (5/5), and *Serratia marcescens* (1/5), and his antibiotic treatment was Daptomycin (500 mg, intravenous daily, 6 weeks) along with levofloxacin (750 mg, oral daily, 6 weeks). He remains fully active without additional issues in the 6 months since (Fig. 2). The transtibial NIC patient had persistent pain, reported subjective micro-motion, and had radiographic evidence of peri-implant lucency. His first additional surgery was unsuccessful attempted removal of the osseointegration implant; it was fixed so sturdily it was considered a greater risk to remove it than to retain it. After 2 more months of symptoms, a second surgery successfully removed the implant without antibiotic depot placement. His index cultures were negative, and the removal cultures grew *Streptococcus agalactiae* (8/8), and his antibiotic management was amoxicillin–clavulanate (875–125 mg, oral daily, 30 d). Nine months after explantation he had revision osseointegration and has been active without issue for more than 1 year.

Four patients had disinfection surgery prior to their subsequent osseointegration. Three had absorbable calcium sulfate antibiotics placed locally, and the fourth had a polymethyl methacrylate cement antibiotic spacer placed which was removed at osseointegration. All four patients had NIC at subsequent osseointegration. One patient was later provided a single 10-day course of doxycycline for minor stoma drainage. The other three patients had no postoperative infectious events.

## 4 Discussion

The most important finding of this study is that UPIC at the time of primary osseointegration does not appear to predispose to an increased risk of additional infection-related management at 1-year-plus follow-up. The UPIC vs. NIC rates of oral antibiotic prescription (2/8 = 25 % vs. 8/22 = 36.4 %, p=0.682 ) and additional surgery to manage infection (1/8 = 12.5 % vs. 1/22 = 4.5 %, p=0.469 ) were not statistically different.

The selection of antibiotics for UPIC in osseointegration has not been directly analyzed before. The delivery of antibiotics to the affected area depends on the vascularity of the local bone and surrounding tissues and is unpredictable in osseointegration cases. This is because amputated bone has been previously traumatized, perhaps more than once, and may be less biologically active since amputees load their amputated residual limb less than their unaffected limb (Bemben et al., 2017). In general, intravenous antibiotics seem to offer no advantage to oral antibiotics for orthopedic infections (Li et al., 2019). Within orthopedics, however, specific indications for the higher concentrations of drug achievable using a parenteral route may exist but remain poorly described. Therefore, in the context of managing UPIC after osseointegration, the effect and optimal choice of antibiotic remains relatively unguided.

An additional consideration is that the unique transcutaneous placement of osseointegration devices poses concerns about infection which go beyond the usual concerns of UPIC. Unlike other surgical reconstructions where the skin is eventually closed, new microbes can presumably enter the body via the stoma and cause infection throughout the lifespan of the transcutaneous osseointegrated device. This study did not evaluate those risks but instead sought to evaluate the risks of UPIC at the time of implantation surgery.

Although no prior osseointegration studies consider the utility of cultures taken during implantation, principles based on the following studies provide context to this investigation and guided our practice. One of the earliest large studies, from 1973 (Fitzgerald et al., 1973), identified 111 positive cultures among 437 (25 %) operation-naive total hip replacements (THRs) and 84 positive cultures among 221 (38 %) previously operated hips. Importantly, they evaluated a subset of 100 patients whose cultures grew “more significant” bacteria. Of 23 patients treated with antibiotics targeted toward the cultured bacteria, none developed wound infections, whereas 5 of 77 patients (6.5 %) who were not provided an antibiotic regimen developed wound infection ( p=0.587 ). Carlsson et al. (1977) identified that routine postoperative empiric prophylactic antibiotic administration decreased total hip infection from 15 % to 2 %. More recently, Picado et al. (2008) reported postoperative infection of 1 in 241 (0.4 %) THRs with zero or one UPIC which received routine postoperative antibiotics, versus 12/22 (55 %) of THRs with two or more cultures despite them receiving targeted extended postoperative antibiotics ( p<0.001 ). A 2006 study identified 4 of 142 (2 %) primary THRs had UPIC; three patients received directed antibiotics and none developed infection (Mehra et al., 2006). A 2014 study found that of 41 UPIC among 90 total hip and knee replacements, there was no difference in implant revision rates (septic or aseptic) through 15 years (Jonsson et al., 2014). In total shoulder replacement, two groups identified very different rates of UPIC but no discernible impact on eventual infection (Zmistowski et al., 2021; Wong et al., 2018; Maccioni et al., 2015). There appears to be low consensus regarding the optimal management of UPIC in revision total joint replacement (Purudappa et al., 2020). Although some literature suggests a single positive culture at revision joint replacement may not require treatment (Neufeld et al., 2021), we prefer to treat even a single positive culture at osseointegration.

Since existing amputees have had prior surgery, there is risk for bacterial contamination seeded at the prior operation. Additionally, osseointegration leaves the intraosseous implant permanently exposed to the outside world, a relatively high risk for colonization to eventually become infection (Kazmers et al., 2016). Admittedly, it is often difficult to differentiate whether mild erythema and non-odorous drainage is due to mere colonization or actual infection. We believe that infection potentially compromising the implant–bone interface may be prevented if the bone can grow onto the implant surface before bacteria do (Hall et al., 1975; Gristina et al., 1988), although with a permanent skin disruption to accommodate the osseointegrated device, it is uncertain whether the antibiotics truly change outcomes (Fragomen et al., 2017). Given the potential high risk of under-treating an bacterial infection that could compromise the implant, versus the relatively low risk of clinically meaningful adverse events associated with a 6-week course of antibiotics featuring close laboratory monitoring (Kokado et al., 2019), we currently choose to provide directed antibiotic augmentation of routine perioperative prophylactic antibiotics for osseointegration patients who have UPIC.

The most notable limitation of this study is the sample size, in particular having only eight UPIC patients. Further, due to sample size, risk factors for UPIC cannot be reliably proposed. Relative strengths of this study are that all included patients had at least 1 year of follow-up with none lost to follow-up, and all culture results and antibiotic plans were fully evaluable. Additionally, interventions are unlikely to have been undocumented since patients were instructed to notify us directly about any infectious concerns and to procure related antibiotic prescriptions only from our team.

## 5 Conclusions

UPIC at the time of primary osseointegration with subsequent antibiotic therapy does not appear to predispose to an increased risk of additional infection-related management versus NIC through 1-year-plus follow-up. Although the therapeutic benefit of providing a course of antibiotics versus no additional antibiotics following UPIC is unknown, it did not appear to increase the risk of other adverse outcomes in our cohort.

## Data Availability

The underlying research data are not accessible because of university and research institution policies.
